# Source Analysis of Beta-Synchronisation and Cortico-Muscular Coherence after Movement Termination Based on High Resolution Electroencephalography

**DOI:** 10.1371/journal.pone.0033928

**Published:** 2012-03-21

**Authors:** Muthuraman Muthuraman, Gertrúd Tamás, Helge Hellriegel, Günther Deuschl, Jan Raethjen

**Affiliations:** 1 Department of Neurology, Christian-Albrechts University, Kiel, Germany; 2 Department of Neurology, Semmelweis University, Budapest, Hungary; University Medical Center Groningen UMCG, The Netherlands

## Abstract

We hypothesized that post-movement beta synchronization (PMBS) and cortico-muscular coherence (CMC) during movement termination relate to each other and have similar role in sensorimotor integration. We calculated the parameters and estimated the sources of these phenomena.

We measured 64-channel EEG simultaneously with surface EMG of the right first dorsal interosseus muscle in 11 healthy volunteers. In Task1, subjects kept a medium-strength contraction continuously; in Task2, superimposed on this movement, they performed repetitive self-paced short contractions. In Task3 short contractions were executed alone. Time-frequency analysis of the EEG and CMC was performed with respect to the offset of brisk movements and averaged in each subject. Sources of PMBS and CMC were also calculated.

High beta power in Task1, PMBS in Task2-3, and CMC in Task1-2 could be observed in the same individual frequency bands. While beta synchronization in Task1 and PMBS in Task2-3 appeared bilateral with contralateral predominance, CMC in Task1-2 was strictly a unilateral phenomenon; their main sources did not differ contralateral to the movement in the primary sensorimotor cortex in 7 of 11 subjects in Task1, and in 6 of 9 subjects in Task2. In Task2, CMC and PMBS had the same latency but their amplitudes did not correlate with each other. In Task2, weaker PMBS source was found bilaterally within the secondary sensory cortex, while the second source of CMC was detected in the premotor cortex, contralateral to the movement. In Task3, weaker sources of PMBS could be estimated in bilateral supplementary motor cortex and in the thalamus.

PMBS and CMC appear simultaneously at the end of a phasic movement possibly suggesting similar antikinetic effects, but they may be separate processes with different active functions. Whereas PMBS seems to reset the supraspinal sensorimotor network, cortico-muscular coherence may represent the recalibration of cortico-motoneuronal and spinal systems.

## Introduction

Initiation, execution, and termination of a movement have a certain temporal spectral evolution [1] in electrophysiological (EEG/MEG) measurements; certain components, which also occur during motor imagery, may assist brain-computer interfaces [2].

Beta rhythm seems to play a special role in information processing of the sensorimotor system. Termination of a sensorimotor task is followed by a central, beta event-related response in the EEG and MEG recordings, called post movement beta synchronization (PMBS) [3], [4]. It principally appears in the region of the primary sensorimotor and supplementary motor cortex [5], [6] but also in premotor areas [7] and secondary somatosensory cortex as a bilateral, contralateral predominant phenomenon [4].

PMBS is a transient, short increase of power 500 to 2500 ms after termination of a movement [3] and occurs in an individual narrow beta frequency band between 15 and 30Hz [8]. Its power and latency depend on the type of the preceding movement [9], [10]. The functional role and central mechanisms of PMBS are still subject to debate. Sensory afferences seem to be necessary for PMBS generation [11], [12]: beta rebound is higher after proprioceptive than after cutaneous inputs [13], underlining the dominant relation of the lemniscal system with PMBS [14]. It is decreased in patients with spinal cord injury [15] and is always connected to the closure of a motor program [16].

A similar PMBS can also be measured in the nucleus subthalamicus (STN) in Parkinson's disease [17], indicating parallel processing of commands both cortically and subcortically in the sensorimotor circuits after executed movement. PMBS in the STN is much lower after movement imagery than after active movement [18]. However, it is not influenced by levodopa intake [17] as PMBS in the cortex; thus these phenomena may not be absolutely identical.

In a previous study, elevated beta synchronization in the sensorimotor cortex was associated with slowing of voluntary finger movement in healthy subjects. It was concluded that high beta activity is associated with maintenance of tonic contractions and impairs the speed of the new movement [19]. Excessive beta activity in the nucleus subthalamicus (STN) is thought to be antikinetic in Parkinson's disease; normal higher frequency synchronization can be reached after levodopa intake [20] or by deep brain stimulation of the STN [21] with improvement of bradykinesia. It is also known that there is excessive beta synchronization in the basal ganglia circuit and the cortex in Parkinson's disease [22], which may be the cause of impaired PMBS in Parkinson's disease [23]. Nevertheless, beta band cortico-muscular coherent oscillatory activity is a physiological phenomenon and seems to be related to normal movement regulation. It is also disturbed in Parkinson's disease, with its frequency shifted to the lower ranges in the “off” state, which is reversible after levodopa intake [24].

Coherence is a measure of the correlation of two signals in the frequency domain. Coherence between EEG/MEG and EMG during weak-to-moderate isometric contractions is also found in the beta frequency band, typically at around 20 Hz [25]–[28], and it disappears when a dynamic movement is initiated. Cortico-muscular coherence reflects the synchronized activity of corticospinal feed-forward and proprioceptive feedback information processing [29]. In the primary motor cortex, it is unilateral for hand movement and follows a somatotopical pattern, and it is bilateral between cortex and axial trunk muscles [30].

The aim of the present study was to investigate whether PMBS and cortico-muscular coherence have a similar functional role in the physiological oscillatory drive of sensorimotor network after movement offset and to define how they are related to each other. We analyzed the parameters and sources of PMBS and cortico-muscular coherence at the same time in a common paradigm.

## Methods

In this study, 11 normal subjects were recruited. All gave written informed consent. The study was approved by the Ethics Committee, Medical Faculty, University of Kiel. Subjects were seated comfortably in an arm chair with their forearms supported on and their hands hanging freely from the armrests. In the first task (Task1), subjects kept a constant medium-strength contraction of the FDI-muscle (constant contraction). In the second task (Task2), superimposed on this contraction they performed a repetitive voluntary self-paced brisk squeeze of the object (a brisk contraction hence, a dynamic task) approximately every 10 seconds. During the third task (Task3), the hands were also supported and the subjects executed only the brisk contraction with complete rest in between. For detection of muscle activity and marking the beginning and the end of brisk voluntary movement, a bipolar surface EMG electrode was placed above the first dorsal interosseus (FDI) muscle. EEG was recorded in parallel with a standard 64-channel EEG recording system (Brain Products Co., Munich, Germany) using a linked mastoid reference. A standard EEG cap was used with electrodes positioned according to the extended 10–20 system. EEG and EMG were band-pass filtered (EMG 30–200 Hz; EEG 0.05–200 Hz) and sampled at 1000 Hz. Data were stored in a computer and analyzed off-line. The EMG was full-wave rectified. The combination of band-pass filtering and rectification is the common demodulation procedure for EMG [31].

The electrode locations covering the primary sensorimotor area, with the highest PMBS power and coherence, were chosen individually contralateral and ipsilateral to the movement (C1–C6) and in the midline (Cz). Rectified EMG signal was used to identify the beginning of the movements, and we signed the EMG and EEG signal at the beginning of the movements with “on” and at the end of the movements with “off” markers. The time delay between the on and off markers was defined as movement duration. We created 8-s-long EEG segments, 4s before and 4s after the off marker position as the time 0. Ocular artifacts were controlled visually at the F1, Fz, and F2 EEG channels. Segments with visible artifacts were manually rejected. Trials were only selected when the beginning and the end of the movement could be clearly defined.

### Time Frequency Analysis

The multitaper method [32]–[34] was used for the spectral analysis. The spectrum was estimated by multiplying the data with 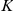 different windows (i.e., tapers). This method uses a sliding time window for calculating the power spectrum by discrete Fourier transformation. If 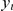 is the signal, then the spectral power is calculated as follows [35]:
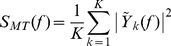
(1)The detailed description of the multitaper method can be found elsewhere [34], [36].

The coherence between the two signals from the healthy subjects, in our case the EEG signal 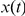 and the EMG signal 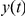, is estimated as follows [37]:
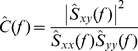
(5)Here 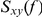 is the cross spectrum and is defined as
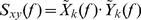
(6)and estimated as given in equation (1). 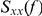, 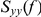 are the individual power spectra, estimated as given in the above equations (1) and (2), the overcap indicating the estimation [38]. The coherence values obtained are normalized between 0 and 1. When the estimated value for the coherence at a frequency is 0, it indicates a lack of correlation between the two signals at this frequency. The value 1 indicates complete correlation between the two signals at this frequency.

In this study, we used windows of length 1000 ms and analyzed the spectral power and coherence within a frequency band of 2–30 Hz. The signals were sampled at 1000 Hz. The time step was 50 ms with overlapping windows and the frequency resolution of 1 Hz. This method has been used earlier in PMBS analysis of patients of Wilson's disease and control subjects [39] and applied to identify sharp changes in power and coherence in Parkinsonian patients [40].

After calculating the absolute power spectra, in each of the three tasks, 35±5 segments were averaged and then baseline corrected with respect to a reference interval from −4 to −3 seconds.

**Figure 1:**
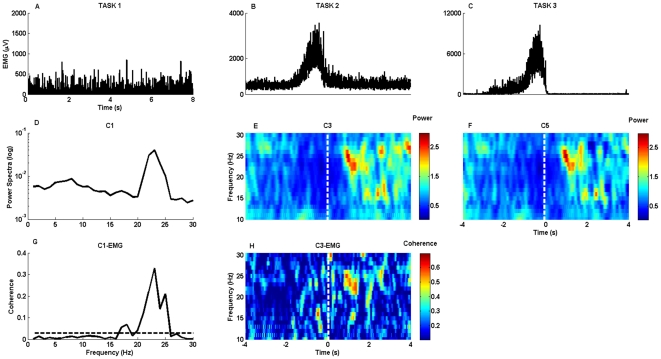
Time-frequency analysis of EEG power and cortico-muscular coherence of one representative subject. The plots A, B and C represent the raw EMG data from the three tasks. The corresponding EEG power spectrums of each task are shown in plots D, E and F. The dashed white line indicates the movement termination in Task2 and Task3. The G shows the coherence between the C1 electrode and EMG in Task1; F plot represent the coherence between the C3 electrode and EMG in Task2. Note the same frequency range for beta power increase and cortico-muscular coherence in the tasks.

PMBS was measured in the most reactive 4-Hz-wide frequency band, which was determined for the strongest contralateral channel for each subject from the time-frequency-relative power plots (Fig. 1). After calculation of relative power values of the most reactive frequency range, we chose the peak power value of beta synchronization (PMBS power) in the 0–2.5s period. The latency of PMBS was determined as the time delay between time 0 and the time when peak PMBS power appeared.

### Statistical analysis

The average duration of the brisk movements in all subjects was compared in the second and third tasks with Wilcoxon matched-pairs test.

The average EMG activity of isometric contraction in Tasks 1 and 2 were compared, as well as the average EMG activity of brisk movements and average EMG activity of isometric contraction measured in Task2, with Wilcoxon matched-pairs test.

To find out if parameters of PMBS depends on movement complexity in different electrode localizations, PMBS power and latency of EEG electrodes contralateral and ipsilateral to the movement and in the midline were compared in Tasks 2 and 3 with repeated measures ANOVA (within factors: TASK, ELECTRODE). This test was also applied separately to compare the average absolute power of the reference interval in these electrode localizations in Tasks 2 and 3 and the average beta power measured during Task1. We used Newman-Keuls post hoc test.

To test if PMBS and CMC appear at the same time and in the same frequency, Wilcoxon matched-pairs test was used to compare their latencies and median frequency values. In the second task, the average power of PMBS, and the amplitude of EMG-EEG coherence of each subject were collected and analyzed with Spearman rank correlation. The level of significance was set to p<0.05.

### Source Analysis

The source analysis used in this study was the dynamic imaging of coherent sources (DICS) [41]–[42] to identify the sources responsible for the individual PMBS frequency band. In order to locate the origin of a specific EEG activity seen on the scalp, two problems need to be solved, which are the forward and inverse problem. The forward problem is the computation of the scalp potentials for a set of neural current sources. It is usually solved by estimating the so-called lead-field matrix with specified models for the brain. In this study, the more complex five-concentric-spheres model was used to create the volume conductor model with standard T1 magnetic resonance images [43]. The template model created was then warped on to the standard head model. The open source software used here is fieldtrip [44]. To map the current dipole in the human brain to the voltages on the scalp, the lead-field matrix (LFM) needs to be calculated [45]. It was estimated using the boundary-element method (BEM) [46]. The head was modeled by inputting the radius and the position of the sphere with the standard electrode locations. The LFM contains the information about the geometry and the conductivity of the model. It defines the projection from current sources at discrete locations in the cranium to potential measurements at discrete recording sites on the scalp.

The inverse problem is the quantitative estimation of the properties of the neural current sources underlying the neural activity. The neural activity is modeled as a current dipole or sum of current dipoles. The power and coherence at any given location in the brain can be computed using a linear transformation, which in this case is the spatial filter [47]. In this study, the linear constrained minimum variance (LMCV) spatial filter was used, which relates the underlying neural activity to the electromagnetic field on the surface. The main aim of the LCMV method [47] is to design a bank of spatial filters that attenuates signals from other locations and allows only signals generated from a particular location in the brain.

Using the time frequency analysis, we identified the reactive PMBS frequency band for each subject as given in Table 1, for which the power estimates were calculated. The frequency of interest was the center frequency of the reactive PMBS band, with a smoothing window of +/−4 Hz. The source analysis was carried out on the basis of the time lock analysis within the time interval of the PMBS between 0 and 2.5s. The source of the strongest power in this frequency band was identified at the first run of the source analysis, and for the next run this source was considered as noise to identify further sources responsible for the frequency band to identifying the network. In the case of the coherent source analysis, the same procedure was followed by taking the EMG as the reference to find the network of coherent sources. The source analysis was carried out on an individual basis and then followed up by a grand average analysis. The individual maps of power and coherence were spatially normalized and interpolated on standard T1-weighted MRI scans. The number of activated voxels was identified for each source in each subject for both power and coherence and was compared with Wilcoxon matched pairs test in Tasks 1 and 2. To analyze the differences between the locations of PMBS and CMC sources, the first source voxel co-ordinates with the maximum PMBS and with the maximum CMC were subtracted. To test the inter-individual variability, the differences were estimated between co-ordinates of the voxel with the maximum power or coherence value and the co-ordinates of the reference voxel (Table 2). The reference voxel was selected from the international consortium for brain mapping (ICBM)-152 atlas for the different sources in each tasks [48], [49]. The matrix of differences between the voxel coordinates was then tested with the Chi-square variance test.

**Table 1:** Topography, frequency and latency of PMBS and cortico-muscular coherence (CMC) contralateral to the movement in Task1-3.

	Topography					Frequency (Hz)					Latency (s)		
No/sex/age (yrs)	Max. Power			Max. CMC		Max. Power			Max. CMC		Max. PMBS		Max.CMC
Task	1	2	3	1	2	1	2	3	1	2	2	3	2
1/F/30	C5	C3	C1	C5	C3	16–20	16–21	15–20	16–20	16–21	2	0.7	2.5
2/M/29	C3	C1	C3	C5	C1	20–24	20–24	20–24	19–23	20–24	0.7	0.5	0.7
3/F/27	C1	C3	C5	C1	C3	21–25	21–25	22–26	21–24	21–25	2.1	1.6	2.1
4/M/40	C5	C3	C3	C1	C3	17–21	18–22	16–20	17–21	18–22	1.8	1.5	1.8
5/M/25	C1	C5	C5	C3	-	16–21	17–21	16–20	16–20	---	2.1	1.7	-
6/F/26	C3	C3	C1	C1	C3	14–19	16–20	15–19	14–18	18–20	0.7	0.7	0.7
7/F/27	C5	C3	C5	C3	C3	16–20	16–20	17–21	16–20	16–20	1.1	0.8	1.1
8/M/31	C1	C1	C3	C3	C1	16–18	15–19	15–19	16–18	16–18	2.1	1.8	1.8
9/M/28	C3	C5	C3	C5	-	17–21	18–22	17–21	17–21	---	1.8	1.5	-
10/M/28	C1	C3	C5	C1	C3	22–25	23–27	23–27	22–26	23–27	0.9	0.6	0.9
11/M/26	C5	C3	C3	C1	C3	19–23	18–22	19–23	19–22	18–22	1.75	1.4	1.75

f: female, m: male.

**Table 2:** MNI co-ordinates of the reference voxels chosen for the sources in each task.

Tasks	Measure	Source number – Brain region	X	Y	Z
1	Power	1- primary sensorimotor hand area	−50	−30	38
1	Coherence	1- primary sensorimotor hand area	−50	−30	38
2	Power	1- motor hand area	−51	−21	43
2	Power	2- Secondary Sensory cortex	−59	−30	28
2	Coherence	1- primary sensorimotor hand area	−50	−30	38
2	Coherence	2-premotor area	−37	−13	38
3	Power	1- primary sensorimotor hand area	−50	−30	38
3	Power	2- supplementary motor area	−12	−21	41
3	Power	3-thalamus	−5	−29	5

## Results

### PMBS and Cortico-muscular Coherence

The average duration of the brisk contraction was longer in Task2 (0.43±0.06s) than in Task3 (0.4±0.07s, p = 0.03).

EMG activity of the constant isometric contraction was higher in Task2 than in Task1 (346.1±321.01 µV and 297.9±287.84 µV, respectively; p = 0.007). In Task2, as expected, EMG activity increased significantly during the brisk movements as compared to the constant isometric contraction (p = 0.002).

EEG beta power during continuous movement in Task1 and baseline beta power in Task2 did not differ (p = 0.99) in the frequency range of 14–26 Hz, but they were significantly higher compared with average baseline beta power in Task3 (p<0.05) in the contralateral central electrodes. In the second and third tasks post-movement beta synchronization was found in all of the subjects on both sides with contralateral preponderance, in locations C1, C3, and C5, in the frequency range of 15–27 Hz.

In comparison of PMBS power in Tasks 2 and 3, the ELECTRODE factor was significant (p<0.05; F_2,20_ = 18.75) owing to typical topography of PMBS in healthy subjects. The TASK factor was also significant (p<0.05; F_1,10_ = 22.53) in all of the electrodes, PMBS power was lower in the second (1.96±1.266) than in the third task (2.24±1.395). We analyzed the average absolute power of the reference intervals (which was taken from −4 to −3 seconds relative to movement offset) in the two tasks. The ELECTRODE factor was significant as a result of higher baseline beta power in the contralateral and midline electrode locations compared with the ipsilateral ones (p<0.05, F_2,20_ = 18.72). The significant TASK factor (p<0.05, F_1,10_ = 27.57) revealed increased baseline beta activity during the second task (0.39±0.253 µV^2^) compared with the baseline activity of the third task (0.11±0.07 µV^2^).

PMBS latency was significantly longer in the second (1.6±0.54s) than in the third task (1.16±0.476s; p<0.05; F_1,10_ = 12.19).

In Task 2, two of the 11 subjects did not show any change in corticomuscular coherence after the brisk squeeze movements. In 9 subjects, the minimum coherence was 0.16 and the maximum coherence was 0.52 in the second task, with an average latency of 1.48±0.65s. However, all of the 11 subjects showed coherence in the first task, and the minimum coherence was 0.12 and the maximum coherence was 0.42. Individual parameters of maximum PMBS and cortico-muscular coherence are summarized in Table 1.

The two subjects having no cortico-muscular coherence in Task2 had the longest movement duration: 55 ms. In these 2 subjects, the difference between the mean EMG activity during constant contraction and the mean EMG activity during the brisk contraction was significantly lower than in all other subjects.

Median frequency values of beta synchronization and corticomuscular coherence were not significantly different in Task1 (p = 0.11) and were the same in each subject in Task2. In the second task, latency of PMBS and the latency of maximum Coherence were not significantly different in the subjects (p = 0.65), showing that these two phenomena appear at the same time (Fig. 1, Table 1). However, the power of PMBS and the maximum values of CMC were not correlated in Task2 (r = −0.53, p>0.05).

### Source analysis

**Figure 2:**
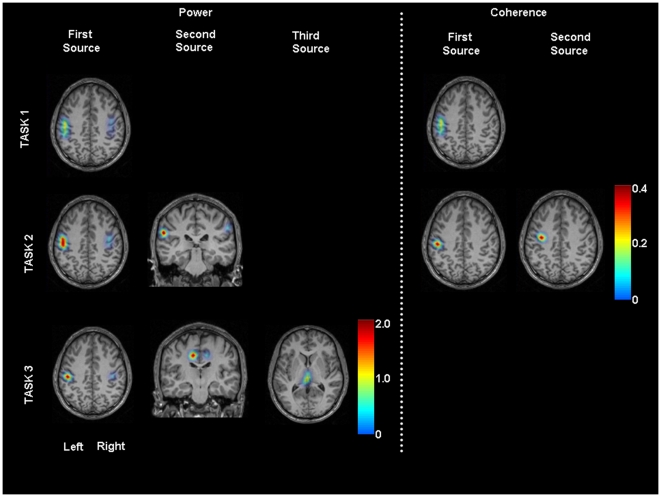
Source analysis of Post-Movement Beta Synchronization and Cortico-muscular Coherence (grand average data). In Task1 high beta power and strong CMC could be detected in the primary sensorimotor cortex. In Task2 the strongest sources could be presented in the same area, but second source of PMBS was estimated in the secondary sensory cortex, second source of CMC in the premotor cortex. Sources of PMBS were bilateral; sources of CMC were unilateral in Task1-2. In Task3 source of PMBS in the primary sensorimotor and supplementary motor area were bilateral and we could detect additional source in the thalamus.

In Task1, the source of high beta activity and the cortico-muscular coherence was in the region of the hand area of primary sensorimotor cortex. The source of PMBS was bilateral and was stronger contralateral to the movement, while the source of the coherence could be seen only on the contralateral side (Fig. 2).

In Task2, the strongest source estimated for the PMBS was identified bilaterally in the hand area of the pre- and postcentral gyrus. The second-strongest source could be seen in the secondary sensory cortex on both sides. In every subject, these sources predominated contralateral to the movement. The strongest source of cortico-muscular coherence was detected in the hand area of the primary sensorimotor cortex, a second source in a premotor area. These sources could be seen only contralateral to the movement in every subject.

In Task3, we calculated again a strong source for PMBS in the hand area of primary sensorimotor cortex, a second source in the supplementary motor cortex, and a third source in the thalamus. The first two sources appeared bilateral and dominated on the contralateral side.

The number of activated voxels was significantly higher for PMBS than for CMC in Task1 (p = 0.000001) and Task2 (first source: p = 0.000006, second source: p = 0.0001) according to paired-comparisons. The first source voxel coordinates (x, y, z) with the maximum amplitude of PMBS and CMC were the same in 7 of 11 subject in Task1 and these coordinates were the same in 6 of 9 subjects in Task2 (Table 3 and 4). There were no significant interindividual differences within the relative voxel coordinates of first source estimated for PMBS and CMC in the three tasks (p>0.05).

**Table 3:** MNI co-ordinates of the first source voxel with the maximum PMBS and maximum coherence in Task 1.

Subjects	PMBS			Coherence		
	X	Y	Z	X	Y	Z
1	−50	−27	38	−50	−27	38
2	−52	−30	38	−52	−30	38
3	−54	−25	38	−52	−23	38
4	−50	−31	38	−50	−31	38
5	−51	−32	38	−49	−30	38
6	−53	−25	38	−53	−25	38
7	−52	−25	38	−52	−25	38
8	−54	−24	38	−52	−22	38
9	−56	−25	38	−53	−22	38
10	−52	−18	38	−52	−18	38
11	−53	−20	38	−53	−20	38

**Table 4:** MNI co-ordinates of the voxels with the maximum PMBS and maximum coherence in Task2.

Subjects	PMBS			Coherence		
	X	Y	Z	X	Y	Z
1	−53	−21	43	−53	−21	43
2	−50	−20	43	−44	−14	43
3	−52	−17	43	−52	−17	43
4	−50	−20	43	−50	−20	43
6	−51	−18	43	−51	−18	43
7	−55	−19	43	−55	−19	43
8	−50	−22	43	−46	−18	43
10	−54	−18	43	−54	−18	43
11	−50	−23	43	−45	−18	43

Note that data of Subjects No 5 and 9 are not in this table because these subjects did not show any change in corticomuscular coherence after the brisk squeeze movements.

## Discussion

In the present study, we investigated the parameters and sources of PMBS and cortico-muscular coherence at the same time with high-resolution EEG and EMG after short self-paced brisk contraction superimposed on constant isometric weak contraction (Task2). The results of this task were compared with those of isometric contraction (Task1) and brisk contractions (Task3) alone. Our aim was to analyze how these phenomena are related to each other and whether movement complexity determines the parameters and sources of PMBS and CMC.

The main findings of this study are as follows:

1. Subjects had the same individual reactive beta frequency band for PMBS and CMC, for both simple and complex movements.
2. PMBS was accompanied by short increase of corticomuscular coherence in the same frequency band, with the same first source of their maximum values in most of the subjects but their magnitudes are not correlated with each other. The first source of PMBS involves bigger cortex area than the first source of CMC.
3. Sources of CMC could be estimated in the primary sensorimotor and premotor cortex, whereas PMBS could be detected in different sensorimotor networks depending on the type and complexity of the task.
4. PMBS could be detected after short movement components during a dynamic, continuous motor task, not only after termination of the whole motor program.

### Parameters of PMBS and cortico-muscular coherence

We could find beta synchronization in an individual frequency band, in each subject after brisk contraction in Tasks 2 and 3, mainly at central contralateral electrodes, where PMBS was previously detected [3]. Increase of cortico-muscular coherence was the highest at the same time, in the same frequency band as of PMBS, in 9 of 11 patients, reflecting active processing between periphery and sensorimotor cortex. This increase could only be seen in the contralateral sensorimotor cortex, with the peak in the sensory cortex.

During simple isometric contraction (Task1), EMG and EEG activity and cortico-muscular coherence were also highest in this beta frequency band (Fig. 1, Table 1). These increases were also observed in studies with healthy subjects performing voluntary isometric contraction [50]–[52].

We have found that the power of PMBS was smaller and its latency was longer in the complex movement (Task2) compared with the brisk movement alone (Task3). The difference in power may be due to the higher baseline power in the complex task. The longer latency may reflect longer processing, e.g. of sensory information, as Task2 definitely needs more precise calibration of muscle strength level; and it may have been influenced by longer movement duration in Task2.

In contrast with previous data suggesting that PMBS only occurs in phases of complete rest [16], [53], we observed PMBS with an ongoing isometric contraction in a complex task consisting of a constant and dynamic phase. During the post-movement beta synchronization, the cortico-muscular coherence was elevated. Since both phenomena appear simultaneously at the end of the brisk phasic movement, they may both be inhibitory phenomena related to blocking a new dynamic phase of the movement, thus being in keeping with an antikinetic effect, as discussed above [19]. On the other hand, the cortico-muscular coherence in the beta band has been hypothesized to reflect recalibration of the motor system after brisk movements [29]. Our data suggest that PMBS may also play a role in this.

In one previous study, EMG coherent EEG activity could be revealed in the beta band at the same time as PMBS during phasic finger movements, though in a narrower frequency band, indicating that PMBS may have a composite nature and that only one of its components may be coherent with EMG activity [7]. Thus, they may simultaneously subserve similar functions in the sensorimotor system, but they do seem to be separate and not mutually dependent processes. We can support this hypothesis with the observations: 1. In Task2, the magnitude of PMBS power was not correlated with the magnitude of coherence and 2. In the same task, we could detect corticomuscular coherence in only 9 out of 11 subjects, but PMBS could be observed in all of them. 3. The cortex area of the first source for CMC was only a part of the first source area of PMBS.

### Sources of PMBS and cortico-muscular coherence

During isometric contraction the source of PMBS and cortico-muscular coherence was estimated in the contralateral primary sensorimotor cortex in accordance with earlier findings [25], [28], [47], [54]. The source of high beta activity during contraction had the same location in most of the subjects, which is in keeping with a role in active processing [12]. In our study the cortex area of first source for PMBS was bigger than the first source area of CMC. This observation again supports the notion that we measured the activity of two sub networks at the same time, after movement termination.

Although it is known that PMBS as measured by surface EEG and MEG largely originates from the sensorimotor cortex, there are a number of hints at other brain regions playing a role in its generation. It was detected predominantly in the precentral region with MEG [55], [56] and EEG [57], although it was dominant in the postcentral gyrus in an EEG-fMRI study [58]. Electrocorticography (ECoG) detected various topographies of PMBS in both the pre- and postcentral gyrus [5], [59]–[60]. PMBS also appeared above supplementary motor cortex [60] and the frontal medial cortex [5]. These results demonstrate that probably several micronetworks exist with beta activity in this area, with different physiological significance [5]. PMBS may reflect the resetting of these micronetworks. This is the synchronized activity of the neurons, which have taken part in the previous movement independent of their role as sensory or motor neurons. The fact that the power of PMBS relates to the parameters of the previous movement (the number of the acting neurons) confirms this theory [9]. In the present study we observed bigger activation size of first source for PMBS in Task2 than in Task3, maybe because more motor and sensory neurons were recruited in Task2.

The source analysis in Task3 of the present study confirms that not only bilateral sensorimotor cortex but also supplementary motor and even subcortical (diencephalic, e.g. thalamic) regions take part in the post-movement beta oscillations. Thus, the postulated micronetworks seem to connect to one large-scale sensorimotor network that is reset after the movement. In the complex movement (Task2), PMBS was not found in the SMA or the thalamus, as in the Task3. Its second source was in the bilateral secondary sensory cortex (SII), with contralateral preponderance indicating that execution of this task is highly related to precise sensory information processing; and in this case, PMBS reflects resetting mainly of sensory networks. It is known that SII has an important role in sensorimotor integration [61] and has higher-order sensory function [4]; active attention to the sensory stimulus increases the activity of SII but not of SI [62].

Conversely, cortico-muscular coherent activity in the beta band was limited to the contralateral side of the movement and only comprised sensorimotor and a premotor areas (Task2). These are the regions where cortico-motoneuron cells have mono-/oligosynaptic connections with the lower motoneurons of the active muscles [63], [64]. The bigger activation size of CMC first source in Task1 compared to Task2 again may reflect that more sensorimotor neurons were involved in Task1.

Thus, the PMBS (beta power) sources are in keeping with a function in a widespread sensorimotor network that seems to be highly task sensitive, possibly reflecting different needs for the kind of information processing. The EMG coherent sources reflect a function of the cortico-muscular coherence, mainly in the corticospinal system. In this study the size of PMBS first source was bigger than the first source of CMC in Task1 and Task2 supporting the more limited nature of the coherent beta activity representing only a part of the more widespread PMBS network.

Our results show that PMBS and cortico-muscular coherence are distinct albeit time-related phenomena at the end of a movement. Whereas it has been postulated that the PMBS resets the supraspinal central components of the sensorimotor network, the cortico-muscular coherent activity may recalibrate the cortico- lower motoneuron and spinal system [29]. It seems that these two functions are not organized in one large coherent network comprising peripheral, spinal and supraspinal centers but in two separate sub networks that typically act simultaneously but independently, thus increasing task-dependent flexibility of post-movement processing in the human motor system.
